# New insight into the additives in preparation and reduction of shield slurry

**DOI:** 10.1038/s41598-023-42939-9

**Published:** 2023-09-25

**Authors:** Zhitao Liu, Silin Wu, Aizhao Zhou, Xiaohui Sun, Haoqing Xu, Shutong Dong

**Affiliations:** 1 School of Architecture and Civil Engineering, Jiangsu University of Science and Technology, Zhenjiang, 212100 China; 2 Jiangsu Province Engineering Research Center of Geoenvironmental Disaster Prevention and Remediation, Zhenjiang, 212100 China; 3 Shenzhen Key Laboratory of Green, Efficient and Intelligent Construction of Underground Metro Station, Shenzhen, 518060 China; 4 College of Civil and Transportation Engineering, The Underground Polis Academy, Shenzhen University, Shenzhen, 518060 China

**Keywords:** Civil engineering, Environmental sciences

## Abstract

In the preparation of the slurry in the slurry shield (SSS) and subsequent reduction of the waste slurry produced by the slurry shield (WSSS), the additives in SSS improve the quality of filtration cake on the excavation surface, but they may also remain in WSSS, which have a negative impact on the reduction efficiency of WSSS. Therefore, it is valuable to establish the relationship between SSS and WSSS with additives as a link. Given this, this paper prepared WSSS with different dosages of additives and studied the influence of residual additives on the reduction. The residual additives made the reduction efficiency of WSSS worse, and the specific resistance to filtration increased by one to two orders of magnitude. The residual additives change the content of bound water or reduce the available sites of the soil particles that can be adsorbed by flocculants, leading to worse reduction results. To reduce the difficulty of reduction, combining polymer and bentonite as additives are recommended to prepare SSS. Polyaluminium chloride (PAC) acts by reducing bound water content through the interaction with residual bentonite, simultaneously augmenting PAM flocculation, which is recommended for reducing WSSS. This paper provides a reference for selecting materials used to prepare SSS and the subsequent reduction of WSSS.

## Introduction

The slurry shield has the advantages of low occupation surface and slight disturbance to the ground, which is widely used in underground engineering, such as subways and tunnels^1,2^. In order to maintain the stability of the tunnel face during the construction of the slurry shield, the slurry in the slurry shield (SSS) is pressurized to form a low permeable filtration cake on the tunnel face^3,4^. Additives such as bentonite and polymer are often added to the SSS to ensure the density and stability of the filtration cake^5–8^. During the excavation process, part of the SSS cannot be recycled, which results in a large amount of waste slurry produced by the slurry shield (WSSS)^9–11^. A small portion of additives may remain in the WSSS, negatively influencing the subsequent reduction. The appropriate additives should improve the quality of the SSS while reducing the negative impact on WSSS reduction. Therefore, it is valuable to establish the relationship between the preparation of the SSS and the reduction of WSSS with (residual) additives as a link.

There are many studies on the influence of additives on the properties of filtration cake. Scholars have proposed that additives can improve the quality of the filtration cake, which improves the stability of the excavation surface^7,12^. However, there are few studies on the impact of residual additives on reducing WSSS. Some studies on reducing other waste slurry might be used as references. Flocculation and filtration were widely used to reduce waste slurry. Flocculants were used to pretreat the slurry so that the fine particles in the waste slurry agglomerate and form flocs, and then the mixture of flocs and slurry was dewatered quickly through filtration. A suitable flocculation pretreatment can significantly improve the efficiency of the reduction^13–15^. Therefore, it is necessary to study the flocculation characteristics of WSSS.

Selecting the appropriate flocculant according to the composition of the slurry was often used in flocculation design. For example, there are components such as extracellular polymeric substances (EPS) and humus in municipal slurry and dredging slurry, which can worsen the dewatering results^16–21^. Given this, some scholars added inorganic coagulants such as polyaluminium chloride (PAC) and polymerized ferrous sulfate (PFS) to the slurry, which reduced the stability of the soil particles, and made the dispersed fine particle in the slurry aggregate into larger flocs, improving the results of flocculation^22,23^. Other scholars used electrochemical Fenton pretreatment to pretreat the slurry, which decomposed the EPS and humus, and released the bound water on the surface of particles, improving the flocculation result^24^. The composition of WSSS is different from municipal slurry and dredging slurry, which may contain residual additives such as bentonite, carboxymethylcellulose sodium (CMC), poly anioniccellulose (PACE), and pregelatinized starch (PGS)^25–27^. There are few studies on the flocculation and filtration of the WSSS. It is necessary to study this content, which can facilitate the reduction of WSSS and further establish the relationship between the preparation of the SSS and the reduction of WSSS.

In this study, different properties of WSSS were prepared with residual additives such as bentonite, CMC, PACE, and PGS. Flocculation and filtration tests were carried out on these WSSS. The influence and mechanism of residual additives on reducing WSSS were discussed. On this basis, a new insight into the preparation and reduction of shield slurry has been proposed, which can provide a reference for selecting materials used to prepare SSS and the subsequent reduction of WSSS.

## Results

### Effect of residual additives on reduction

**Figure 1:**
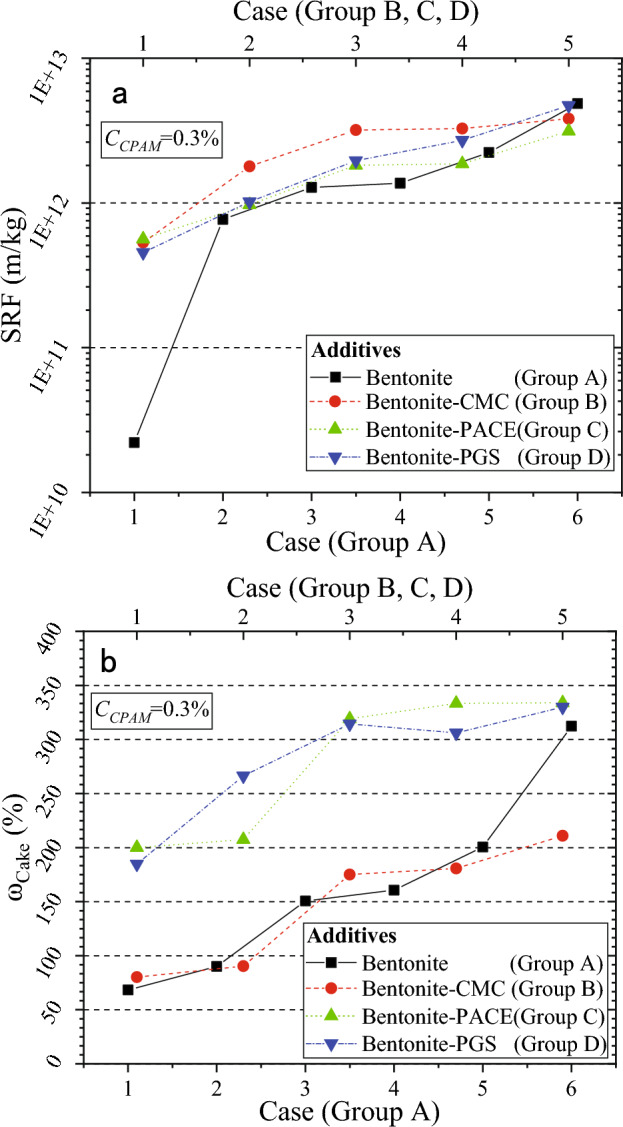
Effect of different additives on dewatering results: (**a**) SRF, (**b**) ω_Cake_.

Figure 1 illustrates the effect of additives on SRF and ω_Cake_. Furthermore, the complete dataset reflecting the variation of filtration volume over time is presented in Supplementary Fig. S3. With the increase of *C*_*Ben*_, *C*_*CMC*_, *C*_*PACE*_, and *C*_*PGS*_, the ω_Cake_ and SRF increased, which indicated that the dewatering efficiency of the slurry became worse. The dewatering results of WSSS with the highest and lowest dosages of additives are summarized in Table 1. The SRF increased by one to two orders of magnitude, and ω_Cake_ increased by two to five times with increased dosages of additives.

**Table 1:** The dewatering results of WSSS with the highest and lowest additive dosage.

Groups	Additives	Cases	Additive dosage (%)	SRF (m/kg)	ω_Cake_ (%)
Group A	Bentonite	A-1	1.0	2.21 × 10^10^	68.28
		A-6	6.0	4.86 × 10^12^	312.30
Group B	Bentonite and CMC	B-1	4.0, 0.4	5.33 × 10^11^	80.20
		B-5	4.0, 2.0	3.82 × 10^12^	211.00
Group C	Bentonite and PACE	C-1	4.0, 1.5	5.64 × 10^11^	200.03
		C-5	4.0, 7.5	3.14 × 10^12^	333.90
Group D	Bentonite and PGS	D-1	4.0, 3.2	4.55 × 10^11^	184.73
		D-5	4.0, 16.0	4.71 × 10^12^	329.92

### Effect of flocculants on reduction

**Figure 2:**
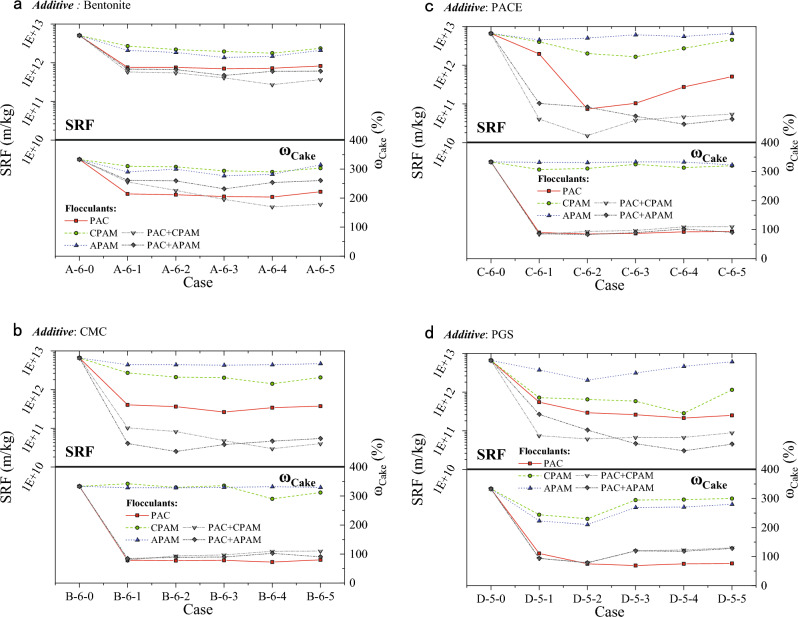
Dewatering results of WSSS with different flocculants: (**a**) bentonite, (**b**) CMC, (**c**) PACE, (**d**) PGS.

Figure 2 illustrates the enhancement in dewatering outcomes of WSSS treated with various flocculants. When subjected to PAM flocculation, the dewatering effectiveness of WSSS displayed marginal enhancement. The SRF remained within a similar range as that of the control group, while the ω_Cake_ surged to a notable 170–364%. Conversely, significant improvement in dewatering efficiency was observed when WSSS underwent flocculation with PAC or dual flocculant combinations. In these cases, the SRF was substantially diminished by an order of magnitude, accompanied by a corresponding reduction in ω_Cake_ to 66%.

Furthermore, a comparison between the dewatering effects of bentonite-based slurry and bentonite-polymer-based slurry revealed the latter’s superior performance. Even after flocculation with PAC or dual flocculants, the SRF of bentonite-based slurry remained within the order of 10^11^ m/kg, with ω_Cake_ surpassing 170%. In stark contrast, bentonite-polymer-based slurry exhibited a remarkable reduction in SRF to the order of 10^10^ m/kg, accompanied by a proportional decrease in ω_Cake_ to approximately 66%.

### Zeta potential

**Figure 3:**
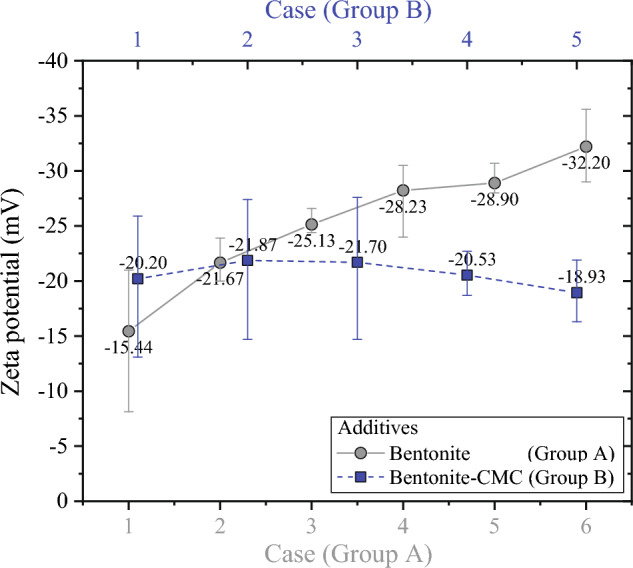
Zeta potential of different types of slurry.

Figure 3 illustrates the impact of *C*_*Ben*_ and *C*_*CMC*_ on the zeta potential of WSSS. Notably, *C*_*Ben*_ has a more pronounced effect on WSSS zeta potential compared to *C*_*CMC*_. The zeta potential of WSSS decreased from − 15.44 to − 32.20 mV as *C*_*Ben*_ increased from 1 to 6%. In contrast, the influence of *C*_*CMC*_ on zeta potential is less significant, with values ranging between − 21.70 and − 18.93 mV.

**Figure 4:**
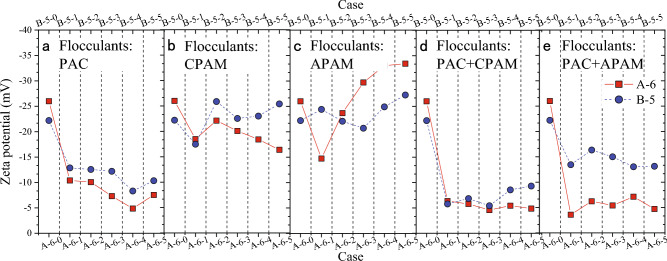
Zeta potential of WSSS treated by different flocculants: (**a**) PAC, (**b**) CPAM, (**c**) APAM, (**d**) PAC + CPAM, (**e**) PAC + APAM.

Figure 4 depicts the influence of diverse flocculation methods on the zeta potential of WSSS. In comparison to PAC or dual flocculants, PAM demonstrates a comparatively minor impact on zeta potential. The introduction of PAC or dual flocculants leads to a substantial elevation in the slurry’s zeta potential, resulting in values ranging from − 4.83 to − 15.42 mV. In contrast, the zeta potential of WSSS treated with PAM remains lower, varying between − 14.72 and − 33.40 mV.

In summary, the zeta potential is subject to varying degrees of influence based on the dosages of residual additives and the choice of flocculants. Concerning residual additives, the impact of bentonite on the zeta potential outweighs that of CMC. As for flocculants, the zeta potential is notably more affected by PAC or dual flocculants than by PAM.

### Soil–water characteristic curve (SWCC)

**Figure 5:**
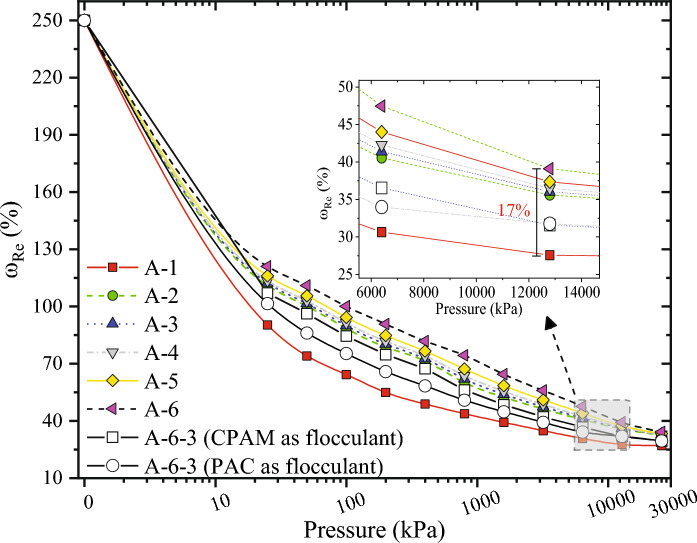
SWCC of bentonite-based slurry.

**Figure 6:**
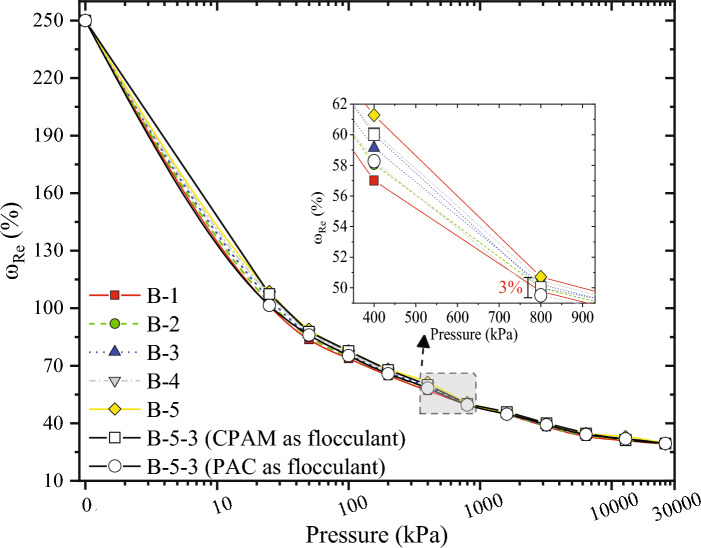
SWCC of bentonite-polymer-based slurry.

Figures 5 and 6 show that the ω_Re_ of the slurry increased with the increase of *C*_*Ben*_ and *C*_*CMC*_. Among them, *C*_*Ben*_ had a more significant effect on ω_Re_ than *C*_*CMC*_. When the *P*_*cen*_ reached 800 kPa, the maximum difference in ω_Re_ between slurry with different *C*_*CMC*_ was only 3%. In contrast, until the *P*_*cen*_ reached 10 MPa, the maximum difference in ω_Re_ between slurry with different *C*_*Ben*_ was still 17%. The decrease in ω_Re_ of the slurry flocculated by PAC was more than that by PAM.

## Discussion

### Mechanism of residual bentonite affecting the dewatering of WSSS

Residual additives lead to poor dewatering results of the WSSS. The discussion begins by elucidating residual bentonite’s mechanistic influence on dewatering performance in the context of WSSS. Figure 3 underscores the substantial reduction in zeta potential with the elevation of *C*_*Ben*_. Zeta potential can characterize the ability of soil particles to absorb water^2^, implying an augmentation in bound water content within the cake due to the residual bentonite present in the WSSS. This augmentation is subsequently confirmed through the SWCC outcomes depicted in Fig. 5, where an increase in residual bentonite correlates with higher values of ω_Re_, reflecting a high bound water content in the cake.

**Figure 7:**
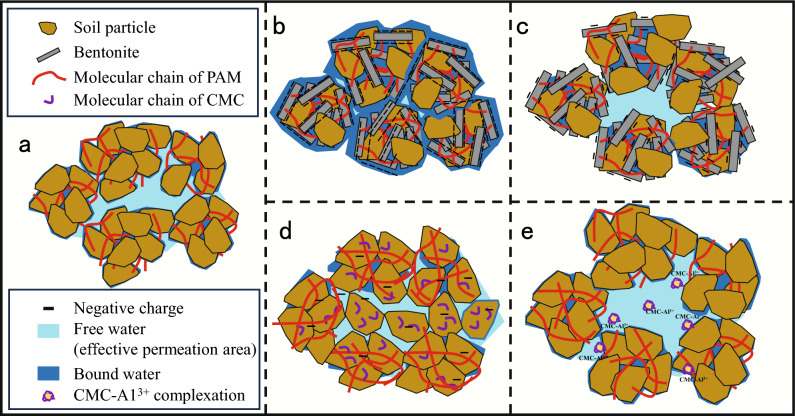
Cross-sectional view of the filtration cake: (**a**) slurry without residual additives flocculated by PAM, (**b**) slurry with residual bentonite flocculated by PAM, (**c**) slurry with residual bentonite flocculated by PAC and PAM, (**d**) slurry with residual CMC flocculated by PAM, (**e**) slurry with residual CMC flocculated by PAC and PAM.

The mechanistic underpinning of residual bentonite’s impact on dewatering is further illustrated vividly in Fig. 7b. Figure 7b presents a cross-sectional view of the cake formation process, portraying a notably high bound water content in the cake containing residual bentonite. The viscous nature of this bound water obstructs certain dewatering channels, resulting in a reduced effective permeation area, consequently leading to compromised dewatering efficiency. In contrast, as depicted in Fig. 7a, cakes devoid of residual bentonite exhibit lower bound water content, facilitating a greater effective permeation area and, subsequently, improved dewatering performance.

The outcomes presented in Fig. 2 unveil a pronounced enhancement in dewatering when PAC or dual flocculants are introduced to the bentonite-based slurry, a phenomenon that can be elucidated in conjunction with Fig. 7c. As discerned from Fig. 4, the zeta potential of the bentonite-based slurry notably increases and tends towards zero upon the addition of PAC or dual flocculants. This observation underscores PAC’s effective reduction in bound water content within the cake, subsequently augmenting the effective permeation area within the cake. Consequently, an improvement in dewatering efficiency is achieved.

### Mechanism of residual polymer affecting the dewatering of WSSS

The mechanistic influence of residual polymer on dewatering warrants discussion. The CMC was used as an example for illustration. Figures 3 and 6 collectively demonstrate that the escalation of *C*_*CMC*_ does not yield a substantial alteration in zeta potential and ω_Re_. This observation suggests that the underlying dewatering mechanism influenced by CMC might differ from that of residual bentonite. When higher levels of *C*_*CMC*_ are present within the WSSS, the use of PAM flocculation results in the formation of smaller flocs, with a median particle size (d_50_) reaching 243.06 μm, as detailed in Supplementary Table S3. Figure 7d provides an illustrative representation of the impact of CMC on dewatering. The reduced effective permeation area within the cake formed by smaller flocs leads to diminished dewatering efficiency.

Conversely, in scenarios where WSSS lacks CMC, the flocculation process yields larger flocs with a d_50_ of 388.76 μm (see Supplementary Table S3). The resultant larger flocs contribute to an increased effective permeation area within the formed cake, consequently facilitating enhanced dewatering performance, as depicted in Fig. 7a.

The findings in Fig. 2 unveil a notable enhancement in dewatering efficiency upon introducing PAC or dual flocculants to WSSS containing residual CMC, an observation expounded through the mechanistic insights derived from Fig. 7e. Both CMC and PAM exhibit adsorption onto soil particles through hydrogen bonding to a certain extent^28,29^. Upon the addition of PAC, complexation occurs between CMC and Al^3+^ within the slurry^30^. This complexation transforms the originally adsorbed CMC on soil particles into a dissolved complex, enhancing the subsequent adsorption of PAM to soil particles introduced into the slurry. As compared to results of floc size without PAC, the addition of PAC raises the d_50_ to 414.26 μm (see Supplementary Table S3). Consequently, PAC facilitates the desorption of CMC from soil particles, enhancing PAM flocculation outcomes and, consequently, amplifying the effective permeation area within the cake. Thus, dewatering performance is improved through PAC’s role in disentangling CMC from soil particles and bolstering PAM flocculation, thereby enhancing effective permeation within the formed cake.

### Strategy for preparation of SSS and reduction of WSSS

The mechanism of residual additives affecting the flocculation and dewatering of WSSS has been analyzed clearly. Based on this, the strategies for the preparation of SSS and the reduction of WSSS were discussed.

#### Strategy for the reduction of WSSS

The addition of PAC yields favorable dewatering outcomes for both WSSS containing residual bentonite and WSSS containing residual polymers, albeit for distinct underlying reasons. Therefore, it is recommended to use PAC when reducing WSSS. If the dewatering efficiency needs to be further enhanced, the combined use of PAC and PAM can be considered.

#### Strategy for preparation of SSS

The dewatering efficiency of bentonite-polymer-based slurry was better than that of bentonite-based slurry under the same flocculation conditions. Therefore, combining polymer and bentonite as additives are recommended to prepare SSS. Since different polymer additives have different effects on the preparation of SSS and the reduction of WSSS, it is necessary to comprehensively analyze and determine the most suitable polymer additive.

Table 2 illustrates the cost of commonly used additives in preparation of SSS and reduction of WSSS and calculates the cost. Although bentonite-based slurry has the lowest cost in preparation of SSS, the total cost of bentonite-based slurry is 17.54–34.40% higher than that of bentonite-polymer-based slurry. Although bentonite-polymer-based slurry has a high cost in the preparation of SSS, when considering the cost of preparation of SSS and the reduction of WSSS simultaneously, it is more economical to use polymer and bentonite as additives. Among them, the total cost of the combination of bentonite and CMC is the lowest and recommended first.

**Table 2:** The total cost ($/t) of additives in preparation of SSS and reduction of WSSS.

Additives in SSS	Preparation of SSS			Reduction of WSSS		Total cost
	Bentonite^a^	Polymer^a^	Preparation cost	PAC^a^	Reduction cost	
Bentonite	7.70	0	7.70	1.95	1.95	9.65
CMC and bentonite	5.24	1.30	6.54	0.65	0.65	7.18
PACE and bentonite	5.24	2.16	7.4	0.81	0.81	8.21
PGS and bentonite	5.24	1.38	6.62	0.81	0.81	7.43

^a^According to local suppliers, the price of bentonite, CMC, PACE, PGS and PAC were 129.38, 1509.42, 1221.91, 431.26, and 229.86 $/t, respectively.

### Limitations

The experimental design entails certain limitations. The employed WSSS was not derived from actual waste but was synthetically prepared by incorporating additives such as bentonite and polymers. The rationale behind not utilizing actual waste lies in the intricate challenge of quantifying residual additive content. In this study’s experimental framework, the WSSS was synthesized to encompass a gradient of additive content. The design of this gradient, as expounded upon in the experimental scheme section, adheres to the principle that additive content does not surpass that found within the SSS.

To validate the outcomes obtained from the artificially formulated WSSS, supplementary tests were conducted using waste slurry generated from a specific pipe jacking project. The results of these additional tests are presented in Supplementary Table S2. These tests affirm the impact of residual bentonite on dewatering within waste slurry and verify the role of PAC.

## Materials and methods

### Materials

**Figure 8:**
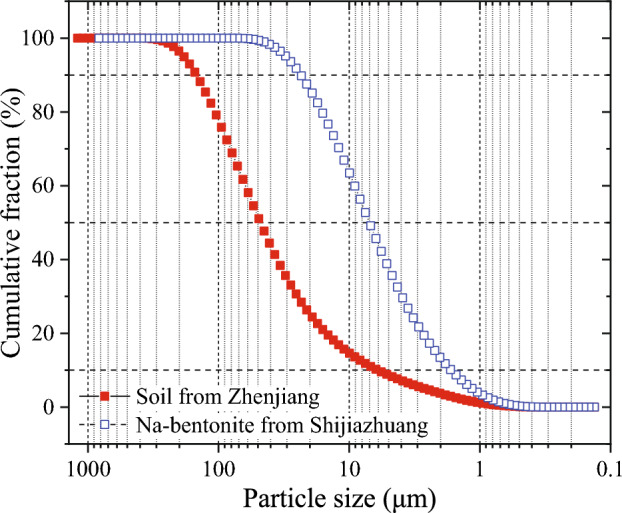
Particle size distribution of soil and bentonite.

The WSSS was prepared by mixing soil, water, and various additives, including bentonite, CMC, PACE, and PGS. The soil was from Zhenjiang, Jiangsu, China, and the bentonite was from Shijiazhuang, Hebei, China. The basic physical parameters of soil and bentonite are shown in Table 3 and Fig. 8. The liquid limit and plastic limit were measured according to ASTM D4318^31^, specific gravity was measured according to ASTM D854^32^, and particle size distribution was measured according to ASTM D4464^33^. Polymer additives such as CMC, PACE, and PGS were produced by Henan Hengrui Starch Technology Co., Ltd., with a viscosity of 2500, 1600, and 1800 cps, respectively.

**Table 3:** The basic physical parameters of soil and bentonite.

Types	Liquid limit (%)	Plastic limit (%)	Specific gravity
Soil from Zhenjiang	42.16	22.18	2.62
Na-bentonite from Shijiazhuang	92.42	30.60	2.64

The flocculants used in the experiments include polyacrylamide (PAM) and polyaluminum chloride (PAC). The cationic polyacrylamide (CPAM) and the anionic polyacrylamide (APAM) were produced by Shanghai Wshine Chemical Co., Ltd., and their molecular weights were 10 million and 12 million, respectively. PAC was produced by Henan Zhongbang Environmental Protection Technology Co., Ltd., and its Al_2_O_3_ content and basicity were about 28% and 75%, respectively.

### Methods

The WSSS, polymer solution, and flocculant solution were prepared, respectively. Subsequently, the WSSS was followed by a flocculation test, filtration test, zeta potential test, and centrifugal test in sequence.

#### Preparation of solution

The dry powder of polymer additives and flocculants was dissolved in tap water and stirred for one hour at 20 °C and 500 r/min. Polymer solutions and flocculant solutions with different mass concentrations were prepared. The mass concentrations of CMC, PACE, and PGS solutions were prepared at 2%, and the mass concentrations of CPAM, APAM, and PAC solutions were prepared at 0.15%, 0.07%, and 3%, respectively.

#### Preparation of WSSS

Soil, bentonite, and polymer solutions were added to tap water proportionally. It was stirred at 20 °C and 500 r/min for 1 h to complete the preparation of WSSS. The water content of the WSSS was prepared to be 250%, and the preparation scheme is shown in Table 4.

**Table 4:** Experiment scheme of WSSS with different additives.

Groups	Cases	Water (g)	Soil (g)	Additives				*C*_*CPAM*_ (%)	Indicators
				*C*_*Ben*_ (%)	*C*_*CMC*_ (%)	*C*_*PACE*_ (%)	*C*_*PGS*_ (%)		
Group A	A-1	300	120	1.0				0.3	SRF, ω_Cake_, zeta potential, ω_Re_
	A-2			2.0					
	A-3			3.0					
	A-4			4.0					
	A-5			5.0					
	A-6			6.0					
Group B	B-1			4.0	0.4				
	B-2				0.8				
	B-3				1.2				
	B-4				1.6				
	B-5				2.0				
Group C	C-1					1.5			SRF, ω_Cake_
	C-2					3.0			
	C-3					4.5			
	C-4					6.0			
	C-5					7.5			
Group D	D-1						3.2		
	D-2						6.4		
	D-3						9.6		
	D-4						12.8		
	D-5						16.0		

#### Flocculation test

The flocculant solution was added to the WSSS and stirred for 5 min at 20 °C and 500 r/min to complete flocculation.

#### Filtration test

To better evaluate the flocculation and dewatering of the WSSS, the specific resistance to filtration (SRF) and the water content of the filtration cake after vacuum filtration (ω_Cake_) were measured. The flocculated WSSS was filtered through a vacuum filtration apparatus, the filtrate was collected, its volume (*V*) was measured, and the corresponding filtration time (*t*) was recorded. SRF was calculated by Eq. (1)^34^. The experimental protocol incorporated either a 30-min filtration period or the occurrence of cake cracking as the termination criteria. Upon meeting either of these conditions, the filtration test was considered concluded^35,36^. The filtration cake was collected, and ω_Cake_ was determined after the filtration test.

1 $${\text{SRF = }}\frac{{2}{\text{PA}}^{2}}{\mu }\times \frac{\text{b}}{{\text{C}}}.$$

SRF is the specific resistance to filtration, m/kg. *P* is the vacuum pressure (60 kPa), kPa. *A* is the filtration area (0.0095 m^2^), m^2^. *μ* is the kinetic viscosity, kg/(m·s). *b* is the slope of the *V* ~ *t*/*V* fitting curve. *C* is the weight of solids intercepted on the filter by filtering a unit volume of filtrate, kg/L.

#### Zeta potential test

To explain the mechanism of residual additives affecting the flocculation and dewatering of WSSS, the zeta potential was measured through electrophoretic light scattering using a Zetasizer Nano ZSP (Malvern Instruments, UK). The zeta potential generally serves as an indicator of the surface charge intensity of soil particles, concurrently reflecting their adsorption capacity for water^2^. By assessing the zeta potential of WSSS, insights into the impact of residual additives on bound water content within the cake can be gleaned. This analytical approach proves instrumental in analyzing the interplay between residual additives and dewatering efficacy.

#### Centrifugal test

The soil–water characteristic curve (SWCC) of WSSS was determined by a high-speed refrigerated centrifuge CR21N (Hitachi, Ltd. Japan). The centrifugal pressures (*P*_*cen*_) were set to 12.5, 25, 50, 100, 200, 400, 800, 1600, 3200, 6400, 12,800, and 25,600 kPa. The corresponding water content of slurry (ω_Re_) dewatered by various *P*_*cen*_ was measured and used to obtain SWCC. SWCC provides a more intuitive depiction of moisture extraction from the cake, serving as a tool to unveil the mechanism through which residual additives impact the flocculation and dewatering processes within WSSS.

### Experimental scheme

#### Residual additives on reduction of WSSS

Four types of WSSS prepared with bentonite, CMC, PACE, or PGS as additives were used to study the effect of residual additives on the reduction of WSSS in Table 4. Including that Group A, bentonite-based slurry with 1.0–6.0% bentonite content (*C*_*Ben*_); Group B, bentonite-polymer-based slurry with 4.0% bentonite content and 0.4–2.0% content of CMC solution (*C*_*CMC*_); Group C, bentonite-polymer-based slurry with 4.0% bentonite content and 1.5–7.5% content of PACE solution (*C*_*PACE*_); Group D, bentonite-polymer-based slurry with 4.0% bentonite content and 3.2–16.0% content of PGS solution (*C*_*PGS*_). The gradient of additive dosages mentioned above is guided by the actual engineering practices of SSS formulation. Considering that the content of residual additives in WSSS cannot exceed the content of additives when preparing SSS, the maximum dosages of bentonite, CMC, PACE, and PGS in Table 4 were set to 6.0%, 2.0%, 7.5%, and 16.0%, respectively^5,37–39^.

0.3% dosage of CPAM (*C*_*CPAM*_) was added to WSSS for flocculation. Then the SRF, ω_Cake_, and other indicators were measured.

#### Flocculants on reduction of WSSS

Different flocculants were used to pretreat WSSS to study the influence of flocculants on the reduction of WSSS, including adding PAC, CPAM, and APAM. Table 5 shows the experiment scheme, including single and dual flocculation. Four kinds of slurry with different additives (A-6, B-5, C-5, and D-5 in Table 4) were selected as WSSS. After flocculation, the SRF, ω_Cake_, zeta potential, and ω_Re_ were measured.

**Table 5:** Experiment scheme of WSSS treated by different flocculants.

Cases	Single flocculation			Dual flocculation^b^		Indicators
	*C*_*PAC*_ (%)	*C*_*CPAM*_ (%)	*C*_*APAM*_ (%)	PAC + CPAM,* C*_*CPAM*_ (%)	PAC + APAM, *C*_*APAM*_ (%)	
A-6-0^a^	No flocculant added					SRF, ω_Cake_, zeta potential, ω_Re_
A-6-1	2.50	0.15	0.070	0.200	0.070	
A-6-2	2.75	0.20	0.105	0.225	0.105	
A-6-3	3.00	0.25	0.140	0.250	0.140	
A-6-4	3.25	0.30	0.175	0.275	0.175	
A-6-5	3.50	0.35	0.210	0.300	0.210	
B-5-0^a^	No flocculant added					
B-5-1	0.50	0.200	0.0475	0.100	0.0475	
B-5-2	0.75	0.225	0.0650	0.125	0.0650	
B-5-3	1.00	0.250	0.0825	0.150	0.0825	
B-5-4	1.25	0.275	0.1000	0.175	0.1000	
B-5-5	1.50	0.300	0.1175	0.200	0.1175	
C-5-0^a^	No flocculant added					SRF, ω_Cake_
C-5-1	0.50	0.200	0.0825	0.100	0.0125	
C-5-2	0.75	0.225	0.1000	0.125	0.0300	
C-5-3	1.00	0.250	0.1175	0.150	0.0475	
C-5-4	1.25	0.275	0.1350	0.175	0.0650	
C-5-5	1.50	0.300	0.1525	0.200	0.0825	
D-5-0^a^	No flocculant added					
D-5-1	0.50	0.200	0.0125	0.100	0.0475	
D-5-2	0.75	0.225	0.0300	0.125	0.0650	
D-5-3	1.00	0.250	0.0475	0.150	0.0825	
D-5-4	1.25	0.275	0.0650	0.175	0.1000	
D-5-5	1.50	0.300	0.0825	0.200	0.1175	

^a^The control cases have no flocculant added.

^b^The dosages of PAC in groups A-6, B-5, C-5, and D-5 were 3%, 1%, 1.25%, and 1.25%.

## Conclusions

The formulation of SSS involves the addition of various agents such as bentonite, CMC, PACE, and PGS to enhance slurry performance. However, the presence of residual additives in WSSS can deteriorate the efficiency of dewatering. As the mechanisms underlying the adverse effects of different residual additives on dewatering vary, WSSS containing distinct residual additives exhibits diverse flocculation and dewatering behaviors. This study conducts flocculation and dewatering tests on WSSS using (residual) additives as mediators, yielding novel insights and conclusions regarding SSS formulation and WSSS reduction:

1. Taking into account the overall cost of SSS formulation, WSSS dewatering, and the impact of different residual additives on WSSS dewatering, it is recommended to incorporate polymer-based additives during SSS preparation.
2. PAC and its combination with PAM are endorsed for efficient WSSS reduction.
3. Residual additives exert varying influences through distinct dewatering mechanisms. The presence of bentonite increases the content of bound water within the filtration cake, while polymer additives weaken flocculation and reduce floc size. Both mechanisms result in a diminished effective permeation area within the cake, leading to compromised water drainage.
4. PAC demonstrates multifaceted optimization for dewatering. It reduces bound water content within the cake and facilitates the desorption of polymers from soil particles. This, in turn, enhances PAM flocculation efficacy and augments the effective permeation area within the cake.

### Supplementary Information

Supplementary Information.

## Data Availability

All data relevant to the study are included in the article or uploaded as Supplementary Information. In addition, the datasets used and/or analyzed during the current study are available from the corresponding author on reasonable request.

## Abbreviations

A: Filtration area
APAM: Anionic polyacrylamide
*b*: Slope of the *V* ~ *t*/*V* fitting curve
C: Weight of solids intercepted on the filter by filtering a unit volume of filtrate
CMC: Carboxymethylcellulose sodium
CPAM: Cationic polyacrylamide
*C*_*APAM*_: Amount of APAM in slurry
*C*_*Ben*_: Amount of bentonite in slurry
*C*_*CMC*_: Amount of CMC solution in slurry
*C*_*CPAM*_: Amount of CPAM in slurry
*C*_*PACE*_: Amount of PACE solution in slurry
*C*_*PGS*_: Amount of PGS solution in slurry
*C*_*PAC*_: Amount of PAC in slurry
d_50_: Median particle size
EPS: Extracellular polymeric substances
*P*: Vacuum pressure
PAC: Polyaluminium chloride
PACE: Polyanionic cellulose
PAM: Polyacrylamide
*P*_*cen*_: Centrifugal pressure
PFS: Polymerized ferrous sulfate
PGS: Pregelatinized starch
SRF: Specific resistance to filtration
SSS: Slurry in the slurry shield
SWCC: Soil–water characteristic curve
*t*: Filtration time
*V*: Filtrate volume
WSSS: Waste slurry produced by slurry shield
*μ*: Kinetic viscosity
ω_Cake_: Water content of cake after filtration
ω_R__e_: Water content of slurry dewatered with various *P*_*cen*_
