# Supplementary material for: New insight into the additives in preparation and reduction of shield slurry
Source: Sci Rep. 2023 Sep 25;13:15969. doi: 10.1038/s41598-023-42939-9 (PMC10519938; doi:10.1038/s41598-023-42939-9)
Supplement: Supplementary file 1 — Supplementary Information. [file 41598_2023_42939_MOESM1_ESM.pdf]

Supplementary Information

New Insight into the Additives in Preparation and Reduction of Shield Slurry

Zhitao Liu[1,2], Silin Wu[1,2,3,*], Aizhao Zhou[1,2], Xiaohui Sun[3,4], Haoqing Xu[1,2], and Shutong Dong[1,2]

[1] School of Architecture and Civil Engineering, Jiangsu University of Science and Technology, Zhenjiang 212100, China

[2] Jiangsu Province Engineering Research Center of Geoenvironmental Disaster Prevention and Remediation, Zhenjiang 212100, China

[3] Shenzhen Key Laboratory of Green, Efficient and Intelligent Construction of Underground Metro Station, Shenzhen 518060, China

[4] College of Civil and Transportation Engineering, the Underground Polis Academy, Shenzhen University, Shenzhen 518060, China

[*] corresponding.author: wusilin@just.edu.cn

1 Verification Experiments of WSSS Dewatering

Actual waste originating from a pipe jacking project in Zhenjiang City, Jiangsu Province, China, was utilized to validate the findings of this study. Two batches of waste slurry were retrieved from the construction site, with a time interval of 80 days between sampling. The properties of the waste slurry are detailed in Table S1, while the particle size distribution is illustrated in Fig. S1. Notably, the liquid limit of Type II waste slurry significantly exceeds that of Type I, accompanied by smaller particle sizes. According to the construction reports of the pipe jacking project, additional bentonite was introduced into SSS during construction, potentially contributing to the elevated liquid limit of Type II WSSS. As such, these two categories of actual waste roughly represent WSSS with varying levels of residual bentonite content.

Table S1 Liquid limit and plastic limit of two types of actual waste

Actual waste	Liquid limit (%)	Plastic limit (%)
Type Ⅰ	35.5	17.5
Type Ⅱ	39.6	20.4

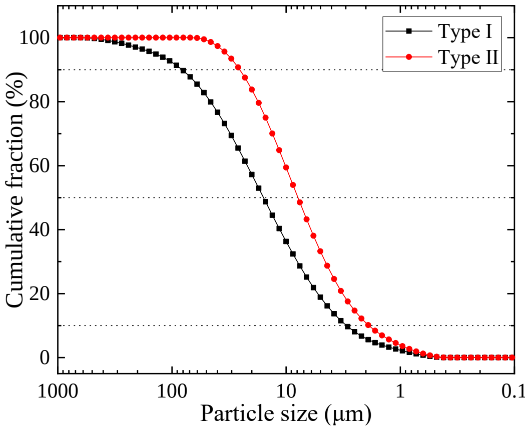

Fig. S1 Particle size distribution of the two types of actual waste

Preparation of the two types of slurry involved formulating them into 250% water content slurry, following the procedure outlined in the main article. Subsequent flocculation and filtration tests were performed, with the experimental protocols and the SRF data documented in Table S2. The experimental methods were consistent with those described in the main article.

Table S2 Flocculation method and SRF

Groups	Cases	Actual waste	Flocculants	SRF (m/kg)
Group E	E-1	Type Ⅰ	C_{CPAM} = 0.15%	9.67×10^{11}
	E-2	Type Ⅰ	C_{PAC} = 3%, C_{CPAM} = 0.15%	6.33×10^{11}
Group F	F-1	Type Ⅱ	C_{CPAM} = 0.15%	2.86×10^{12}
	F-2	Type Ⅱ	C_{PAC} = 3%, C_{CPAM} = 0.15%	7.23×10^{11}

Drawing insights from Table S2 and comparing cases E-1 and F-1, it becomes evident that Type II slurry, characterized by a higher residual bentonite content, exhibits elevated SRF under identical flocculation conditions compared to Type I slurry. This discrepancy indicates a worsened dewatering effect resulting from an increased bentonite content, thereby corroborating the findings of the main article. Furthermore, both slurry types, upon undergoing pretreatment using dual flocculants, demonstrated superior dewatering efficacy compared to single CPAM flocculation, further validating the outcomes presented in the main article.

2 Particle size distribution of WSSS (residual CMC) after flocculation

A portion of the WSSS underwent particle size distribution analysis after flocculation, allowing for an assessment of the flocculation outcomes. The properties of the WSSS and the resulting median particle size (d_{50}) are detailed in Table S3, while the particle size distribution curves are presented in Figure S2. Notably, Case B-1 in Table S3 corresponds to Case B-1 in the main article's Table 4, and Cases B-5-5 (CPAM) and B-5-5 (PAC+CPAM) align with Cases B-5-5 in the main article's Table 5.

From Table S3, it is evident that under similar flocculation conditions, an increase in residual CMC content leads to a significant reduction in d_{50}, diminishing from 388.76 μm to 243.06 μm. Conversely, the addition of PAC results in a noteworthy elevation of d_{50}, increasing it from 243.06 μm to 414.26 μm.

Table S3 Properties of the WSSS and the resulting median particle size

Cases	WSSS				Flocculants		d_{50}
	Water (g)	Soil (g)	C_{Ben} (%)	C_{CMC} (%)	C_{CPAM} (%)	C_{PAC} (%)	(μm)
B-1				0.4	0.3	0	388.76
B-5-5(CPAM)	300	120	4.0	2.0	0.3	0	243.06
B-5-5(PAC+CPAM)				2.0	0.2	1.0	414.26

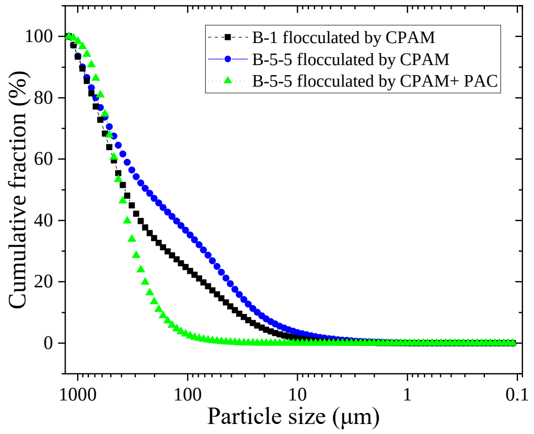

Figure S2 Particle size of CMC slurry after flocculation

3 Filtered volume vs. time curves

Filtered volume vs. time curves for all experiments are shown in Fig. S3.

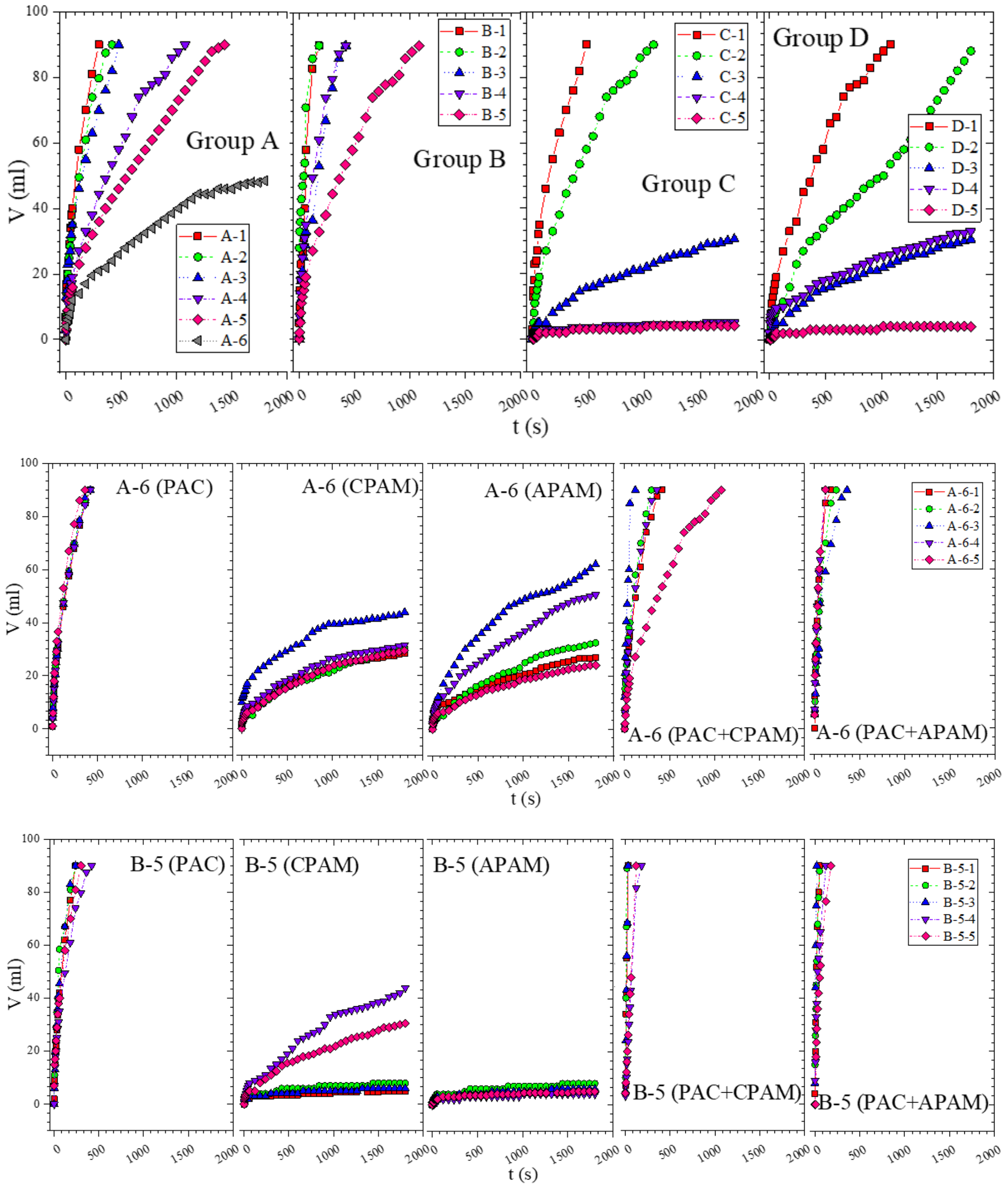

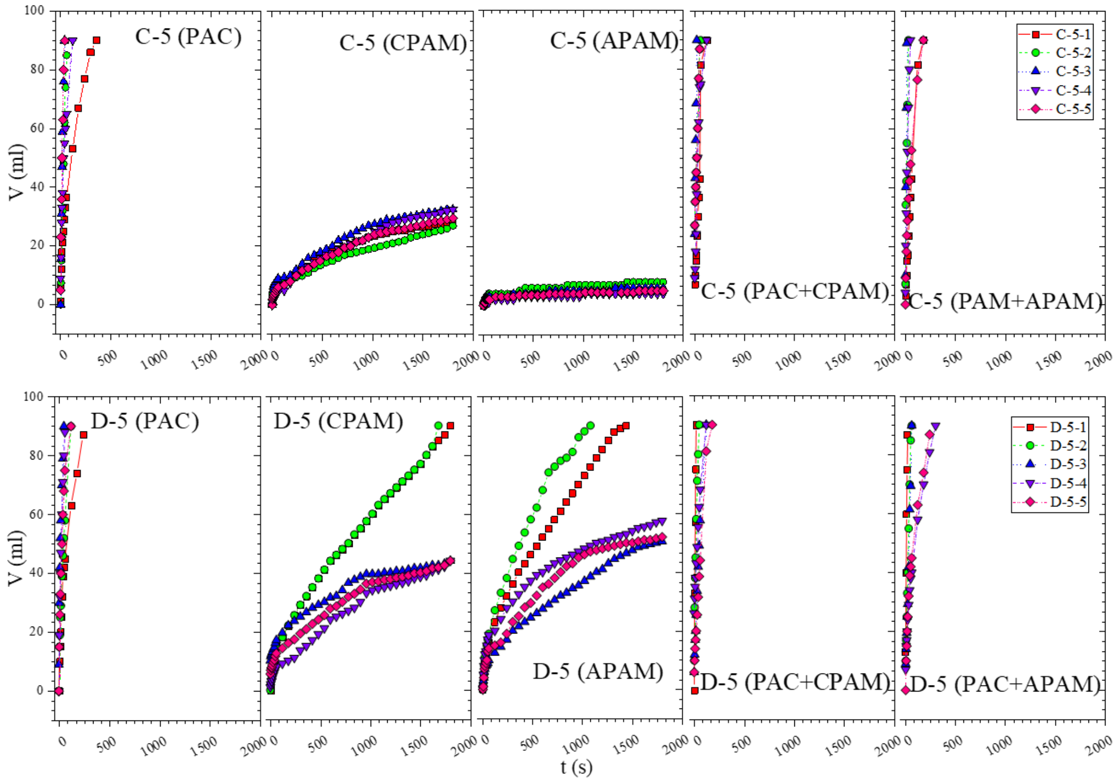

Figure S3 Filtered volume vs. time curves
